# REAMINAS—A Retrospective Study Evaluating the Completeness of the Emergency Department’s Admission Medication and Its Influence on Discharge Medication

**DOI:** 10.3390/jcm15103902

**Published:** 2026-05-19

**Authors:** Ludwig vom Hofe, Maximilian Günther, Daniela Huttner, Ute Amann, Jan Rémi, Matthias Klein, Dorothea Strobach

**Affiliations:** 1 Doctoral Program Clinical Pharmacy, LMU University Hospital, LMU Munich, 81377 Munich, Germany; 2 Hospital Pharmacy, LMU University Hospital, LMU Munich, 81377 Munich, Germany; 3 Faculty of Medicine, LMU Munich, 81377 Munich, Germany; 4 Department of Neurology, LMU University Hospital, LMU Munich, 81377 Munich, Germany; 5 Emergency Department, LMU University Hospital, LMU Munich, 81377 Munich, Germany

**Keywords:** medication reconciliation, pharmacist, unintended medication discrepancy, emergency department, discharge, high-risk medication

## Abstract

**Background**: Documentation of admission medication is frequently insufficient, particularly in the emergency department (ED). This study analyses discrepancies between the admission medication by ED physicians and pharmaceutical medication reconciliation (PMR) at the emergency admission ward, their clinical relevance, and influence on discharge medication. **Methods**: In a retrospective observational study (May 2022–April 2023) on an interdisciplinary emergency admission ward, unintended medication discrepancies (UMDs) between ED admission medication and PMR, as well as prescription discrepancies (PDs) between admission medication prescribed at the emergency admission ward and PMR, were analysed. Persistence of PDs up to discharge was evaluated. Drugs associated with discrepancies were classified for clinical relevance during hospitalisation as (A) relevant for documentation and prescription, (B) relevant for documentation but irrelevant for prescription, or (C) irrelevant for documentation and prescription. Additionally, a list of high-risk drugs was established. **Results**: For 256 patients, a median of three (Q1–Q3 0–6.75) and five (2–8) drugs were documented as admission medication in the ED and PMR, respectively, with a median of two (1–5) UMDs per patient. For Group A drugs, the admission medication prescribed at the emergency admission ward compared to the PMR resulted in a median of one (0–3) PD per patient. Drug omission was most common (60.0 and 61.0% of UMDs and PDs, respectively). A total of 22.8% of UMDs and 23.8% of PDs concerned high-risk drugs. Of 215 PDs eligible for discharge analysis, 137 (63.7%) persisted up to the discharge letter. **Conclusions**: A considerable number of discrepancies were found between the admission medication in the ED and PMR. A substantial proportion of these were caused by high-risk drugs, highlighting their potential to harm patients. Discrepancies tend to persist throughout hospitalisation up to the discharge letter.

## 1. Introduction

The transition from outpatient to inpatient care and vice versa is an error-prone process. Accurate documentation of patients’ pre-hospital medication at hospital admission (admission medication) is crucial to ensure drug therapy safety in the hospital [1]. Studies have shown that up to 64% of prescribing errors in hospitals occur at admission [2,3], leading to inadequate continuation of pre-hospital medication and preventable drug-related problems during hospitalisation, as well as inadequate discharge medication [1,4]. Drug-related problems are all events or circumstances involving drug therapy that actually or potentially interfere with desired health outcomes [5].

Admission via an emergency department (ED) presents particular challenges in terms of correct documentation of pre-hospital medication, given the stressful environment for physicians and patients. This problem is exacerbated by the frequent absence of patients’ medical records, patients’ inability to provide information about their admission medication due to serious medical conditions, and the unavailability of patients’ general practitioners at night and on weekends. Consequently, patients’ admission medication documented in the ED is often inaccurate and incomplete [6,7]. This not only reduces drug therapy safety in the hospital but also prevents the detection of drug-related hospital admissions, which are a major cause of unplanned admissions and death in hospital [8]. The electronic patient file, which should contain all medical information of a patient from ambulatory and hospital care, was introduced in Germany in 2025. However, as of January 2026, the number of active users remains low, suggesting a limited impact on these issues.

The emergency admission ward is part of the emergency department and primarily receives patients from the ED who require further acute diagnostics, treatment or monitoring. While many of its patients can be discharged quickly (usually within 24 h), others require a longer hospital stay and are transferred to different wards. Although admission via the ED has previously been shown to be prone to errors in the admission medication [6,7,9], the risk of these errors resulting in patient harm and incorrect medication during hospitalisation and in discharge letters has not yet been thoroughly investigated. Importantly, not all admission medication errors have the potential to cause patient harm, making an assessment of their clinical relevance necessary. Previous studies, mostly conducted in medical and surgical wards, found 11–59% of admission medication errors to be of clinical relevance [10].

Medication reconciliation (MR) is an important tool for reducing medication errors at transition of care. It is defined as “[…] the formal process in which health care professionals partner with patients to ensure accurate and complete medication information transfer at interfaces of care.” [11]. Studies have shown that, compared to physicians or other healthcare staff, medication reconciliation performed by a pharmacist (PMR) significantly improves the accuracy and completeness of the admission medication documentation [12,13]. Therefore, PMR is an excellent way to ensure the admission medication is documented accurately and completely at the transition of care, especially in critical settings such as the emergency department and emergency admission ward.

Previous studies have examined medication discrepancies occurring during transition of care [3]. However, these focused more on the number of discrepancies than on their clinical impact, which depends on the associated drug and the relevance of its continued prescription. Therefore, we conducted an in-depth analysis on discrepancies between the admission medication documented by physicians in the emergency department or prescribed at the emergency admission ward and the admission medication derived from the pharmacists’ medication reconciliation in the emergency admission ward. Furthermore, to emphasise the importance of this critical step, we aimed to determine the influence of these discrepancies on discharge medication and to evaluate their relevance for the subsequent ambulatory care. Analysing discrepancies at the drug level will enable physicians in the emergency department to identify which drugs are most likely to be associated with discrepancies and which of these are most likely to cause patient harm.

## 2. Materials and Methods

### 2.1. Study Setting and Design

The emergency admission ward at Campus Großhadern of the University Hospital of LMU Munich, a tertiary care hospital in Munich, Germany, is an interdisciplinary intermediate care unit for patients requiring neurological, internal medicine or surgical care. The emergency admission ward receives most of its patients from the ED. Patients are most commonly admitted due to the need for further inpatient (intermediate care) monitoring, acute diagnostics or therapy after ED admission, but also due to the lack of available beds in the hospital’s regular wards, particularly at night. Patients are usually discharged or transferred within one day. At the ED, physicians document the patients’ pre-hospital medication. When patients are admitted to the emergency admission ward, this medication is manually transferred into the hospital’s electronic medication record and prescribed.

PMR was implemented at the emergency admission ward in April 2022 for patients present during the pharmacist’s working hours, carried out according to national and international guidelines [14]. All prescription and non-prescription drugs, herbal products and dietary supplements used prior to admission are documented based on patient interviews as well as information obtained from previous hospital stays, general practitioners, nursing homes or relatives, when appropriate. A modified version of the hospital’s standardised drug history questionnaire is used for the patient interviews. The resulting admission medication is screened for drug-related problems such as necessary dose adjustments for renal or hepatic insufficiency and drug–drug interactions. It is then compared with the medication prescribed by the ED physician in the hospital’s electronic prescribing software. The pre-hospital medication and recommendations for adjusting the prescribed medication regarding discrepancies and drug-related problems are communicated to the attending physicians via the electronic prescribing system. In the event of the patient being discharged or transferred prematurely, these are documented in paper format.

The monocentric retrospective observational study REAMINAS was conducted among adult patients at the Großhadern emergency admission ward who were admitted via the ED. All patients admitted between 1 May 2022 and 30 April 2023 with documented PMR from the emergency admission ward were included.

### 2.2. Outcomes

The primary outcome of the study was the number and type of discrepancies between the admission medication according to the PMR and:

(1) The admission medication according to the ED’s admission report, resulting in unintended medication discrepancies (UMDs).
(2) The prescribed admission medication at the emergency admission ward, resulting in prescription discrepancies (PDs).

**Figure 1:**
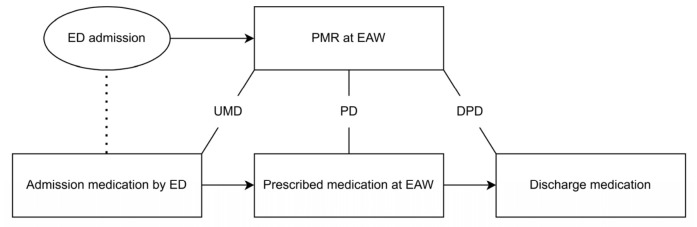
Discrepancy flow chart. ED: emergency department; PMR: pharmaceutical medication reconciliation; EAW: emergency admission ward; UMD: unintended medication discrepancy; PD: prescription discrepancy; DPD: discharge prescription discrepancy.

The secondary outcome was the number of PDs identified on admission that persisted throughout hospitalisation up to the discharge letter (discharge prescription discrepancies, DPDs) (Figure 1). PDs were considered resolved by the PMR if the relevant medication was adjusted by a physician in the electronic prescribing software during the stay on the emergency admission ward or within two days after transfer to another ward. Resolved PDs were not considered for the discharge letter analysis. Additionally, patients discharged on the day of admission or the day after were excluded from the discharge letter analysis, as it was unlikely that the associated discrepancies would be resolved within such a short period.

### 2.3. Discrepancy Classification

The medication discrepancy taxonomy published by Almanasreh et al. [15] was adapted to categorise the discrepancies, resulting in the following seven types: drug omission, drug commission, strength, frequency, drug form (only considered when relevant for therapy), pausing, and (unspecified) active ingredients.

To account for differences in the clinical relevance of UMDs and PDs, the drugs associated with discrepancies were classified into three groups:

Group A: relevant for documentation on admission and in-hospital prescription (e.g., most prescription drugs).
Group B: relevant for documentation on admission, irrelevant for in-hospital prescription (e.g., herbal supplements with potential for interaction).
Group C: irrelevant for documentation on admission and in-hospital prescription (e.g., homoeopathics).

The classification was conducted following a modified Delphi method [16,17]. Using the online tool Welphi (Decision eyes, Lisbon, Portugal), an expert panel consisting of two physicians and three pharmacists assessed all documented drugs in two rounds. If there was 80% agreement (four out of five experts) or more on a drug, it was placed in the respective group. Any drugs for which no agreement was reached after the second round were discussed by the panel to reach a consensus.

As Group C drugs were considered irrelevant in the context of the study, only Group A and B drugs were considered for UMDs. As Group B drugs were irrelevant for in-hospital prescription, only Group A drugs were considered for PDs. Unless the active ingredients were independently affected by discrepancies, medications containing two or more active pharmaceutical ingredients were counted as a single drug.

### 2.4. High-Risk Medication

To assess the potential risk of patient harm arising from the discrepancies, we developed a list of medicines with significant risk to cause patient harm in transition of care. Drugs from published lists of high-risk medicines by Saedder et al. [18] and Doerper et al. [19] were combined and adapted by the study team. After a review of the remaining non-high-risk drugs documented for the study, two pharmacists and one ED physician of the study team additionally deemed some of these to be high-risk, particularly in an ED setting. This was based on the clinical impact of medication discrepancies regarding these drugs or their unintended discontinuation. These drugs were therefore added to the compiled list. The complete list of high-risk drugs can be seen in Table A1.

### 2.5. Data Collection and Handling

Medical data were obtained from the electronic patient information system (SAP-i.s.h.med, Cerner Corporation, North Kansas City, MO, USA) and the computerised physician order entry–clinical decision support system (CPOE-CDSS) Meona (Mesalvo GmbH, Freiburg, Germany). Medication data were obtained from Meona, ED admission reports, hospital discharge letters and the PMR records. The data were processed using Excel 2021 (Microsoft Corporation, Redmond, WA, USA). Statistical analysis was conducted using Excel 2021 for basic analysis and SPSS Version 29 (IBM, Armonk, NY, USA) for in-depth analysis. DeepL Write Pro (DeepL, Köln, Germany) was used for language editing during the preparation of this manuscript.

### 2.6. Statistical Analysis

Descriptive statistics were used to analyse the data. Medians and quartiles (Q1 and Q3) were calculated for numerical data. Categorical data are presented as absolute and relative frequencies. Given the exploratory nature of this study and the descriptive analysis of the data, it was deemed unnecessary to consider sample size calculation.

## 3. Results

### 3.1. Delphi Process

The Delphi process for classifying drugs occurring in this study into the three groups according to their clinical relevancy initially included 117 drugs and drug groups (items). There was no agreement on 60 items after round 1, which decreased to 33 items after round 2. During the subsequent discussion, the experts were able to categorise 24 of the remaining 33 items. The remaining nine items were agreed to be assessed on a case-by-case basis, at the discretion of two pharmacists, depending on the respective patient’s clinical situation.

### 3.2. Patient Characteristics

A total of 256 patients were included in the study. Their demographic characteristics are shown in Table 1. The most prevalent admission diagnoses in the ED were intracranial injury (*n* = 20), cerebral infarction (*n* = 17) and epilepsy (*n* = 15). The majority of patients were subsequently transferred to other wards of the hospital (*n* = 185; 72.3%) or discharged (*n* = 48; 18.8%).

With a median Emergency Severity Index (ESI) of 3 (Q1–Q3 2–3, range 1–5), most patients were stable in the ED but required multiple types of resources for acute diagnostics and treatment. Patients of all urgency levels were included in the study. Of the 196 patients with a documented Glasgow Coma Scale (GCS) score, the median score was 15 (15–15, 5–15), indicating that most had no impairment of consciousness. Most patients (*n* = 230; 89.8%) could be interviewed for the PMR. Other or additional sources had to be consulted for 155 patients (60.5%).

### 3.3. Admission Medication According to the PMR

The analysis of the admission medication according to the PMR resulted in a median of five documented drugs per patient (Q1–Q3 2–8, range 0–27). Of the 1435 drugs, 1114 were classified as Group A drugs (relevant for documentation and in-hospital prescription) and 321 as Group B drugs (relevant for documentation, but not in-hospital prescription). Because of their classification as irrelevant for documentation and in-hospital prescription, 126 Group C drugs were excluded from all further analysis.

Table 2 shows the most frequently documented drugs in Groups A and B. Of the documented Group A and B drugs, 298 (26.8%) and 58 (18.1%) were classified as high-risk, respectively. The most frequently documented high-risk drugs were acetylsalicylic acid (ASA) 100 mg (*n* = 44) and apixaban (*n* = 34) in Group A, and ibuprofen (*n* = 34) and ASA 500 mg (*n* = 5) in Group B.

### 3.4. Unintended Medication Discrepancies

**Figure 2:**
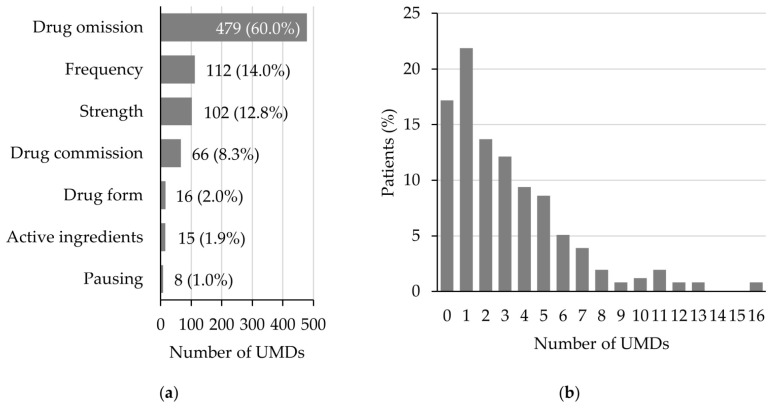
UMDs at admission to the emergency admission ward. (**a**) Distribution of UMDs by type; (**b**) distribution of UMDs per patient. UMD: unintended medication discrepancy.

To evaluate the documentation quality of the ED’s admission medication, it was compared to the admission medication derived from the PMR. For the 256 patients, a median of three (Q1–Q3 0–6.75) drugs per patient were documented as admission medication in the ED, compared to a median of five drugs per patient documented in the PMR. This resulted in a median of two UMDs per patient (Q1–Q3 1–5, range 0–16) in 212 of the 256 (82.8%) patients (Figure 2b). Of the 798 identified UMDs, the most prevalent was drug omission (479 UMDs; 60.0%), followed by discrepancies in frequency (112 UMDs; 14.0%), and strength (102 UMDs; 12.8%) (Figure 2a).

**Figure 3:**
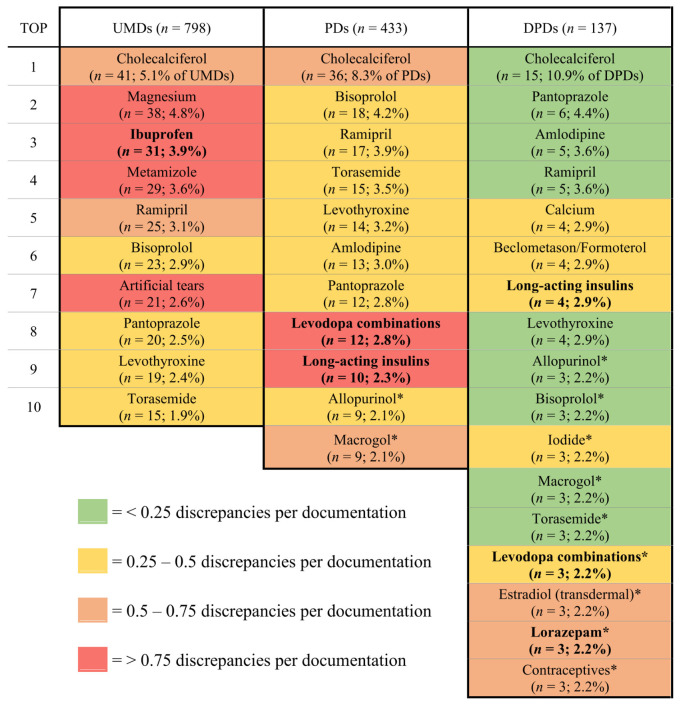
Most frequently documented drugs associated with UMDs, PDs and DPDs. UMD: unintended medication discrepancy; PD: prescription discrepancy; DPD: discharge prescription discrepancy. Bold items are high-risk drugs. * Equal number of documentations in 9th and 10th place.

The analysis of the drugs associated with these UMDs (Figure 3, left column) revealed that six of the ten most prevalent drugs were Group A drugs (cholecalciferol, ramipril, bisoprolol, pantoprazole, levothyroxine and torasemide), indicating their relevance for documentation and in-hospital prescription. The UMD-to-documentation ratios (an indicator of documentation quality) for these drugs ranged from 0.39 (torasemide) to 0.65 (cholecalciferol) UMDs per documentation of the respective drug, suggesting moderately good documentation quality in the ED. In contrast, the documentation quality for the remaining four Group B drugs (magnesium, ibuprofen, metamizole and artificial tears) was poor, with UMD-to-documentation ratios exceeding 0.75 for each drug.

Of the 798 UMDs, 182 (22.8%) were caused by high-risk drugs, with ibuprofen being the most prevalent (31 UMDs), followed by levodopa combinations (10 UMDs), apixaban, ASA 100 mg and long-acting insulins (8 UMDs each). As the only high-risk drug in the top 10, ibuprofen was documented poorly in the ED (UMD-to-documentation ratio of 0.91). While apixaban and ASA 100 mg were frequently documented high-risk drugs, their UMD-to-documentation ratios were low (0.24 and 0.18, respectively), suggesting high documentation quality in the ED.

### 3.5. Prescription Discrepancies (PDs)

**Figure 4:**
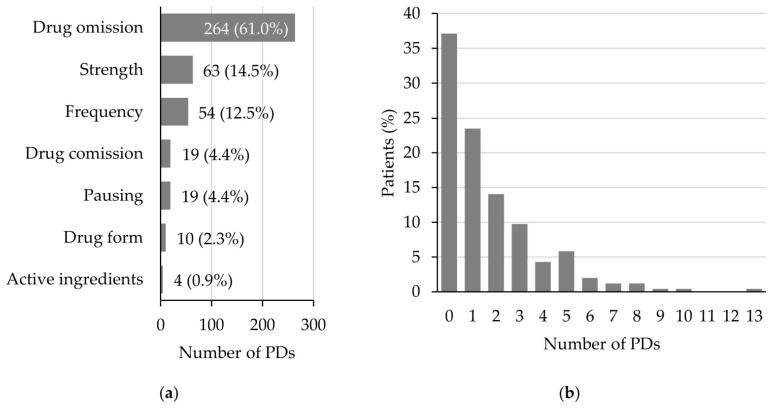
PDs at admission to the emergency admission ward. (**a**) Distribution of PDs by type; (**b**) distribution of PDs per patient. PD: prescription discrepancy.

Having identified a high number of UMDs in the ED’s documentation of the admission medication, we further evaluated the quality of prescription of the admission medication in the emergency admission ward. To assess this, the admission medication according to the PMR was compared with the admission medication prescribed in the electronic prescribing software at the emergency admission ward (prescription discrepancies, PDs). Only Group A drugs were included in this analysis, since Group B and C drugs were irrelevant for in-hospital prescription. A median of one PD per patient (Q1–Q3 0–3, range 0–13) was found in 161 of the 256 (62.9%) patients (Figure 4b). The most common PD was drug omission (264 PDs; 61.0%), followed by discrepancies in strength (63 PDs; 14.5%) and frequency (54 PDs; 12.5%), as shown in Figure 4a.

The drugs most commonly associated with these 433 PDs are shown in Figure 3 (centre column). The PD-to-documentation ratios of these drugs ranged between 0.25 and 0.75, except for levodopa combinations and long-acting insulins.

Interestingly, levodopa combinations and long-acting insulins were also the only high-risk drugs in the top ten. With one of the highest PD-to-documentation ratios (1.20), levodopa combinations were among the drugs with the poorest prescription quality. Long-acting insulins also had a poor prescription quality, as evidenced by their high PD-to-documentation ratio of 0.77. Finally, apixaban and ASA 100 mg, which were two frequently documented high-risk drugs with a high documentation quality in the ED, once again had a relatively high prescription quality, with PD-to-documentation ratios of 0.21 and 0.09, respectively. Overall, 103 of the 433 (23.8%) PDs were caused by high-risk drugs.

### 3.6. Discharge Prescription Discrepancies (DPDs)

**Figure 5:**
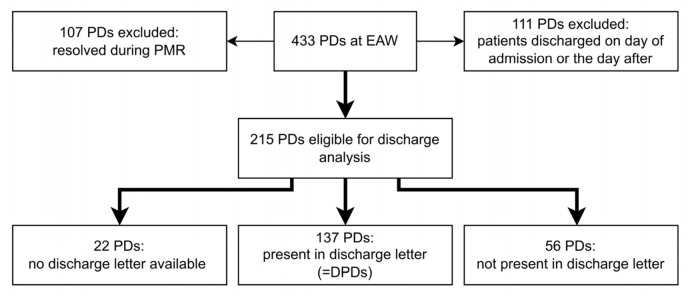
Discharge analysis flowchart. PMR: pharmaceutical medication reconciliation; EAW: emergency admission ward; PD: prescription discrepancy.

As incorrect discharge medication can have a significant influence on therapy and safety, the extent to which PDs at admission persist throughout hospitalisation is of high interest. To analyse this, the patients’ discharge letters were examined for the identified PDs. As shown in Figure 5, of the 433 PDs documented in 161 patients in the emergency admission ward, 107 PDs in 53 patients were resolved during the PMR. This left 326 PDs in 140 patients that remained unresolved after the PMR. Of these patients, 40 (111 PDs) were excluded from the discharge letter analysis because they were discharged on the day of admission or the day after. Of the remaining 215 PDs in 100 patients, 137 (63.7%) in 78 patients were found in the discharge letter as DPDs, 56 PDs (26.0%) in 33 patients were not present in the discharge letter, and for seven patients (22 PDs, 10.2%), no discharge letter was available because they died during hospitalisation.

**Figure A1:**
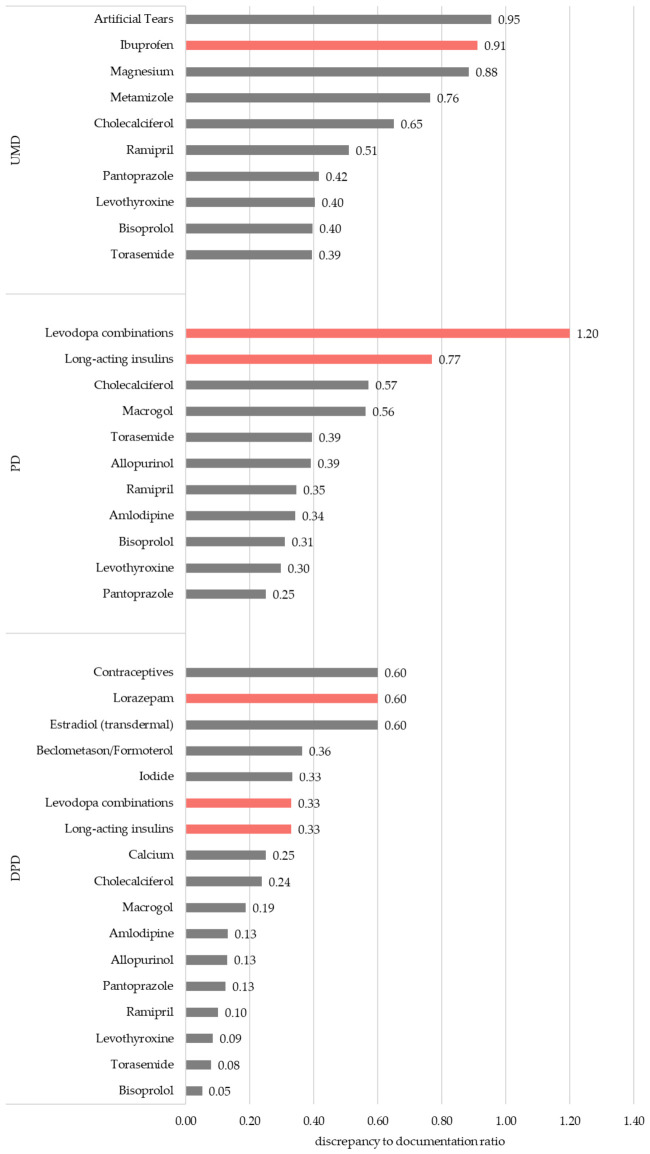
Discrepancy-to-documentation ratios of the most frequently documented drugs associated with UMDs, PDs and DPDs. UMD: unintended medication discrepancy; PD: prescription discrepancy; DPD: discharge prescription discrepancy. Red bars indicate high-risk drugs.

With 84 discrepancies (61.3%), drug omission was the most prevalent DPD type, followed by discrepancies in strength (20, 14.6%) and frequency (19, 13.9%). The most common drugs associated with the 137 identified DPDs are shown in Figure 3 (right column). As a substantial number of PDs were already resolved during the PMR and hospitalisation, the prescription quality of drugs in the discharge letter was higher than in the emergency admission ward, with DPD-to-documentation ratios mostly below 0.25. Of the 215 unresolved PDs at the emergency admission ward, 158 (73.5%) were also identified as UMDs at admission, as were 109 of the 137 (79.6%) DPDs. The collected discrepancy-to-documentation ratios of the three discrepancy types can be found in the Appendix A (Figure A1).

Of the 137 DPDs, 26 (19.0%) were associated with high-risk drugs. The most common high-risk drugs associated with DPDs in the discharge letter were long-acting insulins (four DPDs), levodopa combinations and lorazepam (three DPDs each).

## 4. Discussion

This study characterised in detail the medication discrepancies that occur at hospital admission via the ED, focusing on their clinical relevance in documentation and prescription as well as their persistence up to discharge.

In a cohort of 256 patients, we found a median of two UMDs per patient for drugs classified as relevant for documentation in 83% of patients. Drug omission was by far the most prevalent discrepancy, accounting for 60% of total UMDs, suggesting relevant under-documentation of admission medication at the emergency admission ward. Furthermore, regarding only drugs classified as relevant for prescription in the hospital, we found prescription discrepancies for 63% of patients with a median of one PD per patient. Finally, initially documented PDs were detected in the discharge letters of 78 of the 100 patients eligible for analysis. Of these 137 DPDs, 80% were also documented as UMDs at the emergency admission ward, suggesting that incorrect documentation at the beginning of hospitalisation led to these discharge prescription discrepancies.

High-risk drugs, as adapted from the literature, accounted for 23% of all UMDs, 24% of all PDs and 19% of all DPDs. This study shows that medication discrepancies occur at hospital admission via the ED and highlights the clinical relevance of this issue by categorising discrepancies according to their relevance for documentation or prescription. Additionally, it demonstrates that medication discrepancies often persist from admission via the ED until the patient’s discharge, thereby posing an ongoing risk to drug therapy safety and efficacy.

Several studies have reported medication discrepancies at admission via the ED, with a wide range of 1.7–9.7 discrepancies per patient, concerning 59–88% of admissions [20]. However, the focus should be on clinical relevance rather than on the number of discrepancies alone. Therefore, we took a new approach by categorising documentation and prescription relevance in a Delphi procedure with an interprofessional panel of physicians and pharmacists. Drugs deemed irrelevant for documentation (Group C) were excluded from all further analysis, as they were not expected to be medically relevant.

Even when Group C drugs were excluded, our study found that ED physicians documented a median of three drugs per patient compared to a median of five documented by the pharmacist at the emergency admission ward, resulting in a median of two UMDs per patient. Several drugs in the pre-hospital medication regimen are not prescribed in hospital. However, it is important to be aware of their intake, as they may cause adverse drug reactions or interfere with further diagnostics or therapy (Group B).

Two examples of the most prevalent drugs causing UMDs in our study are ibuprofen and metamizole. Ibuprofen increases the risk of gastrointestinal bleeding, impairs renal function and may interfere with ASA for inhibition of platelet aggregation [21]. Accordingly, ibuprofen is classified as a high-risk drug [18,19]. The actual risk to the patient depends on the dosage and frequency of ibuprofen intake, as well as additional factors such as concomitant medication and underlying diseases. Therefore, questioning the patient should cover aspects of their current usage. Metamizole is a well-known cause of agranulocytosis and a moderate inhibitor of cytochrome P450 enzymes, resulting in clinically relevant drug interactions [22].

Group A consists of drugs that are important for documentation as well as prescription and includes most prescription-only drugs. However, long-term prescription drugs such as antihypertensives and levothyroxine are often not documented and prescribed properly, which increases the risk of hypertensive episodes or hormonal imbalances. Notably, with above-average documentation quality, ED physicians paid particular attention to drugs associated with an increased risk of bleeding, such as apixaban or ASA 100 mg.

By contrast, low prescription quality was found for long-acting insulins and levodopa combinations. Incorrect or missing insulin therapy can cause hyper- or hypoglycaemia, which can easily result in patient harm. In addition, since patients often continue to administer their own insulin in the hospital independently of attending physicians’ and nurses’ orders, incorrect or missing prescriptions in the electronic prescribing software can lead to potentially life-threatening hypoglycaemic events.

Incorrect levodopa therapy can cause serious problems for patients with Parkinson’s disease, whereas the consequences for those with restless legs syndrome may be less severe. Several discrepancies regarding levodopa combinations occurred during the prescription process, but not during documentation. This may be due to the wide variety of strengths and forms available, which makes it harder to prescribe correctly compared to other drugs.

The new framework categorising drugs into Groups A, B and C presented here can guide physicians in the ED to pay special attention to drugs or drug groups especially important for documentation and prescription. Our study also confirmed the beneficial effect of pharmacists performing medication reconciliation in the ED setting, as described in the literature [20,23]. Other advantages reported in the literature include time savings for physicians, lower costs, reduced length of stay in the ED and improved identification of adverse drug reactions (ADRs) [7]. For instance, one study found that 6.5% of all ED visits were accounted for by ADRs [24]. A detailed analysis of patients presenting with ADRs in the ED revealed that 93% had at least one error in their medication list obtained by the physician, and 51.3% of the drugs involved in ADR were associated with an error in the patient’s medication history [7].

Apart from the occurrence of UMDs and PDs at admission, we analysed whether PDs would persist throughout hospitalisation, up to and including the discharge letter. We found that a significant number of PDs were still present in the discharge letter, including those involving high-risk drugs. Similar findings have been reported for patients admitted to geriatric wards, with up to 41% of discrepancies from admission appearing in the discharge letter, though an additional PMR reduced discrepancies [4]. This highlights the importance of accurately documenting pre-hospital medication upon admission, as discrepancies tend to persist throughout the entire hospital stay. Although the clinical impact of these discharge discrepancies has not yet been thoroughly investigated, it is conceivable that general practitioners will incorporate them into patients’ long-term medication plans. These changes have the potential to cause serious patient harm, particularly with regard to high-risk drugs. Therefore, preventing discharge discrepancies seems an important way of increasing drug therapy safety. Further research, particularly regarding the outpatient effects of these discrepancies, is highly desirable.

According to a recent review, the general prevalence of medication errors in the ED is around 23%, with up to 50% occurring during prescription. Failure to accurately identify all pre-admission medications is one key contributor [25]. A study by Lorsbach et al. found that 59% of the patients admitted via the ED had no medication list or letter from their general practitioner. However, additional information could be retrieved later for 48% of patients [26]. In our study, most patients on the emergency admission ward were eligible for questioning, as indicated by a high GCS score. Nevertheless, the pharmacist consulted a median of one additional source to compile a comprehensive pre-hospital medication list. Importantly, Lorsbach et al.’s study found that, had full information been available initially, the diagnostics or therapy of 9% of patients would have needed to be changed [26].

The classification of drugs as “high-risk” has been established and adapted to the setting of an emergency department [18,19]. High-risk drugs were common among drugs causing UMDs and PDs in our study, highlighting the clinical impact of a structured MR at admission.

This study has several limitations. Due to the retrospective nature of the study, patients were not actively enrolled but were included based on the presence of a documented PMR. Therefore, only patients present at the emergency admission ward during the pharmacist’s working hours were included in this study. While this does not mean that enrolment was limited to patients admitted during certain times of the day, it is possible that our approach produced selection bias. Combined with the monocentric nature of our study and the size of our study population, this may affect the generalisability of our findings.

Although the Delphi method is a viable, systematic approach to achieving expert consensus, there is a risk of subjectivity bias, especially with a small expert panel. However, an interprofessional panel, such as the one in our study, should provide a comprehensive range of opinions. Of course, our classification of drugs in terms of their relevance for documentation and prescription may not be directly applicable to other settings. Unlike UMDs, which are by definition not intended by the documenting physician, prescription discrepancies in this study were not verified as being unintended by the prescribing physicians. Although the study team thoroughly reviewed the patients’ prescription documentation in hospital and rejected discrepancies with indicators of intent, there is a possibility that some prescription discrepancies were unrecognised intentional discrepancies. This could lead to an overestimation of the number and clinical influence of PDs. However, correct classification as intended or unintended would have been easier with a more thorough documentation in the ED and emergency admission ward regarding medication changes like pausing or discontinuation. In addition, the retrospective design prevented the documentation of data that were missing in the first place. As the transfer of the PMR into the CPOE had only been fully implemented in July 2022, 97 PDs were only documented but not communicated to the attending physician. This meant that these discrepancies could not be resolved through the PMR. Similarly, 34 PDs were not communicated to the physician because the patients were quickly transferred or discharged.

This study identified discrepancies in transition of care that could potentially cause harm to patients. Although we recognised the need to determine the risk factors associated with these discrepancies, this was not possible in our retrospective study due to missing data. We are therefore currently investigating potential risk factors associated with discrepancies in a prospective study.

## 5. Conclusions

Unintended discrepancies between the pre-hospital medication and the documented admission medication in the ED have the potential to be of high clinical relevance. While some drugs only need to be documented in order to fully assess the patient’s situation, others have to be prescribed continuously during hospitalisation. Discrepancies occurred in both groups and have the potential to negatively influence patient outcomes. Moreover, discrepancies tend to persist until the patient is discharged, thereby posing an even greater risk for drug therapy safety and efficacy. The results of this study suggest that ED physicians should pay closer attention to NSAID and levodopa combinations when interviewing patients. Conducting an additional PMR in the emergency admission ward improved the assessment of pre-hospital medications.

## Figures and Tables

**Table 1:** Patient characteristics (*n* = 256).

	*n* (%)	Median (Q_1_–Q_3_; Range)
Male sex	144 (56.3)	
Age (years)	256	65 (53–80.75; 19–98)
ESI	256	3 (2–3; 1–5)
GCS	196 (76.6)	15 (15–15; 5–15)
eGFR (mL/min/1.73 m^2^)	256	
<30	14 (5.5)	
30–60	40 (15.6)	
>60	202 (78.9)	
Transfer	256	
Discharge from hospital	48 (18.8)	
Internal transfer	185 (72.3)	
Transfer to external hospital	19 (7.4)	
Deceased	4 (1.6)	
Patient interview possible	230 (89.8)	
Days on emergency admission ward	256	1 (1–2; 0–13)
Medical specialty	256	
Surgery	87 (34.0)	
Neurology	59 (23.0)	
Internal medicine	110 (43.0)	

ESI = Emergency Severity Index; GCS = Glasgow Coma Scale; eGFR = Estimated Glomerular Filtration Rate (2021 CKD-EPI Creatinine).

**Table 2:** Most frequently documented Group A and B drugs.

TOP	Group A (*n* = 1114)	Group B (*n* = 321)
1	Cholecalciferol (*n* = 60; 5.4%)	Magnesium (*n* = 43; 13.4%)
2	Bisoprolol (*n* = 58; 5.2%)	Metamizole (*n* = 38; 11.8%)
3	Ramipril (*n* = 49; 4.4%)	**Ibuprofen (*n* = 34; 10.6%)**
4	Levothyroxine (*n* = 47; 4.2%)	Artificial tears (*n* = 22; 6.9%)
5	Pantoprazole (*n* = 47; 4.2%)	Vitamin B12 (*n* = 13; 4.0%)
6	**ASA 100 mg (*n* = 44; 3.9%)**	Paracetamol (*n* = 11; 3.4%)
7	Amlodipine (*n* = 38; 3.4%)	Folic acid (*n* = 8; 2.5%)
8	Torasemide (*n* = 38; 3.4%)	Vitamin B complex (*n* = 8; 2.5%)
9	**Apixaban (*n* = 34; 3.1%)**	Omega-3 fatty acids (*n* = 6; 1.9%)
10	Atorvastatin (*n* = 29; 2.6%)	ASA 500 mg (*n* = 5; 1.6%) *
		Calcium (*n* = 5; 1.6%) *
		Sodium chloride (inhalation) (*n* = 5; 1.6%) *

ASA = acetylsalicylic acid. Bold items are high-risk drugs. * Equal number of documentations in 10th place.

## Data Availability

The data presented in this study are available on reasonable request from the corresponding author due to data privacy.

## Abbreviations

The following abbreviations are used in this manuscript:

ED	Emergency department
PMR	Pharmaceutical medication reconciliation
EAW	Emergency admission ward
UMD	Unintended medication discrepancy
PD	Prescription discrepancy
DPD	Discharge prescription discrepancy
ESI	Emergency Severity Index
GCS	Glasgow Coma Scale
eGFR	Estimated Glomerular Filtration Rate (2021 CKD-EPI Creatinine)
ASA	Acetylsalicylic acid
ADR	Adverse drug reaction
CPOE-CDSS	Computerised physician order entry–clinical decision support system

## Appendix A

**Table A1:** High-risk drugs. The table presents the list of high-risk drugs/drug groups and the respective reference; for additions by the authors, the reason for inclusion is also given.

Medication	Reference	Reason for Inclusion by Authors
Antidiabetic drugs		
**Insulins**	[18,19]	
**Biguanides**	[19]	
**Sulfonylurea**	[19]	
**Glinides**	[19]	
Antithrombotic agents		
**Vitamin K antagonists**	[18,19]	
**Other oral anticoagulants**	[18]	
**Antiplatelet agents**	[19]	
**Heparins**	[19]	
Cardiac therapy		
**Cardiac glycosides**	[18,19]	
**Amiodarone**	[18]	
Propafenone	[18]	
Flecainide	[18]	
**Corticosteroids for systemic use**	[18]	
Methotrexate	[18]	
**Antineoplastic agents per os**	[19]	
**Azole antimycotics**	1	High interaction potential
Erythromycine	[18]	
Rifampicin	[18]	
**Antiretrovirals**	[19]	
**Immunosuppressants**	[19]	
**Systemic NSAID**	[18]	
**Colchicine**	1	Narrow therapeutic index
**Opioids**	[18,19]	
**Antiepileptics**	[18]	
MAO-A-Inhibitors	[18]	
Psycholeptics		
**Antipsychotics**	[18]	
Lithium	[18]	
**Benzodiazepines**	[18]	
**Z-drugs**	1	Fall risk
Psychoanaleptics		
**Tricyclic antidepressants**	[18]	
**Methylphenidate**	1	High risk of severe side effects
**SSRI**	1	Bleeding risk, serotonin syndrome
**SNRI**	1	Bleeding risk, serotonin syndrome
**St. John’s wort**	1	High interaction potential
**Levodopa combinations**	1	Frequent incorrect documentation in ED
**Dopamine agonists**	1	Narrow therapeutic index
**Thalidomide and analogues**	1	High risk of severe side effects

[18] = Saedder et al.; [19] = Doerper et al.; 1 = added by the study authors. Bold drugs are present in the study population.
